# Expression of CCR8 is increased in asthma

**DOI:** 10.1111/j.1365-2222.2010.03504.x

**Published:** 2010-08

**Authors:** K Mutalithas, C Guillen, C Raport, R Kolbeck, D Soler, C E Brightling, I D Pavord, A J Wardlaw

**Affiliations:** 1 Department of Infection, Immunity and Inflammation, Institute for Lung Health, University of LeicesterLeicester, UK; 2 ICOS CorporationBothel, WA, USA; 3 MedimmuneGaithersburg, MD, USA; 4 Millennium PharmaceuticalsCambridge, MA, USA

**Keywords:** asthma, CCR8, chemokine receptor, lung T cell

## Abstract

**Background:**

Chemokines and their receptors could play key roles in the recruitment of T cells to the asthmatic lung. CCR8 is preferentially expressed on T-helper type 2 cells, and is thought to play a role in the pathogenesis of human asthma.

**Objective:**

Determine the expression of CCR8 on T cells in blood, bronchoalveolar lavage (BAL) and bronchial mucosa from asthmatics and normal subjects.

**Methods:**

CCR8 expression in blood and BAL from asthma and normal subjects was studied using flow cytometry. CCR8 expression on IFN-γ^+^ and IL-4^+^/IL-13^+^ blood and BAL T cells was studied following stimulation with Phorbol–Myristate–Acetate and Calcium Ionophore. Paraffin-embedded bronchial biopsies were used to study CCR8 in bronchial epithelium.

**Results:**

The percentage of CD3^+^ cells expressing CCR8 in the blood was higher in asthmatics (4.7±0.4%) compared with normal subjects (3.0±0.4%; *P*<0.01). There was an approximately sixfold enrichment of CCR8 on IL-4^+^/IL-13^+^ cells compared with IFN-γ^+^ T cells (*P*<0.001) in both asthmatic and normal subjects in both blood and BAL. Significantly more BAL T cells expressed CCR8 in asthmatic (8.6±0.8%) compared with normal subjects (3.9±0.7%) (*P*<0.01). In paired blood-BAL samples from asthmatics, significantly more CCR8+CD3^+^ T cells were present in BAL (9.0±0.9%) than in blood (5.6±0.9%; *P*<0.05). There were more CCR8-positive cells in bronchial biopsies from asthmatic (93±11 cells/mm^2^) compared with normal subjects (30±16 cells/mm^2^) (*P*<0.05). The ligand CCL1 was increased in the BAL of asthmatics compared with normal subjects (35±6 vs. 12.9±7 pg/mL; *P*<0.05).

**Conclusion:**

There may be a role for CCR8 in the recruitment of T cells to the lung in asthmatics.

*Cite this as*: K. Mutalithas, C. Guillen, C. Raport, R. Kolbeck, D. Soler, C. E. Brightling, I. D. Pavord and A. J. Wardlaw, *Clinical & Experimental Allergy*, 2010 (40) 1175–1185.

## Introduction

Asthma is a disease characterized by chronic inflammation of the airways [1]. T cells are thought to play a crucial role in initiating and maintaining this inflammatory process [2–7]. Within the family of T cells are a number of subsets covering a wide range of functions. A T-helper type 2(T_H_2) predominant inflammatory process initiated through the cytokines IL-4, IL-5, IL-9 and IL-13 is thought to be central to asthma pathophysiology [8–11]. Understanding the molecular mechanisms by which the various T cell subsets are recruited to the asthmatic airway would help design future therapeutic strategies for asthma.

Increased numbers of T cells are seen in the bronchial mucosa of asthmatics following an allergen challenge, and this increase in numbers has been shown to be mostly due to recruitment from blood [12, 13]. The movement of T cells between the vascular compartment and tissues is carefully co-ordinated during homeostasis and in disease through the concerted expression of adhesion molecules and chemokine receptors [14, 15]. Chemokines, together with their G-protein-coupled receptors, may be involved in organ-specific homing of T cells. For example, CCR4 and CCR9 expression was shown to target T cells to the skin and gut, respectively, in a mutually exclusive manner [16, 17]. Human lung T cells have a unique chemokine receptor profile, which is CXCR3^high^, CCR5^high^, CXCR6^high^, cutaneous lymphocyte antigen (CLA)^−^, and CCR9^−^ [18].

The T_H_1 and T_H_2 subsets of lymphocytes express a restricted repertoire of chemokine receptors *in vitro*. T_H_2 cells preferentially express CCR3, CCR4 and CCR8, while T_H_1 cells express CXCR3 and CCR5 [19]. Ligands for CCR3 and CCR4 have been shown to be increased within the asthmatic airway following allergen challenge [20, 21]. However, we were unable to demonstrate a corresponding increase of T cells expressing CCR3 or CCR4 among bronchoalveolar lavage (BAL) T cells in stable mild asthmatics [22]. CCR8, whose only host ligand is CCL1, is expressed on a subset of CCR4-expressing T_H_2 and regulatory T cells [23, 24]. Animal models of asthma have provided conflicting data on the role of CCR8 in allergic inflammation. Three studies have suggested that the receptor is not involved in mediating allergic inflammation in the mouse model of asthma [25–27]. However, T cell recruitment, T_H_2 cytokine production and mucus production were inhibited in CCR8-deficient mice using either a mast cell-dependent protocol or a model of fungal asthma [28, 29]. The limited studies in humans have also been conflicting. One group found exceptionally high levels of expression of CCR8 in asthmatic sub-mucosa, whereas another found very low levels of expression in biopsies, with no difference from control subjects. In contrast, using *in situ* hybridization, a modest increase in the numbers of CCR4 T cells expressing CCR8 in the bronchial mucosa of asthmatics compared with normal subjects was observed [28, 30]. Similarly, two studies have found an increase in CCL1 in asthma, whereas others have not [28, 30–32].

One problem in studying CCR8 expression in human tissue has been the paucity of good reagents. We have used two reagents, a highly selective monoclonal antibody to CCR8 developed by ICOS Corporation and fluorescently labelled CCL1 (Millennium Pharmaceuticals), to study the involvement of CCL1 and its receptor CCR8 in asthma. Our data support a possible role for CCR8 in the recruitment of T_H_2 cells to an asthmatic lung.

## Materials and methods

### Subjects

Thirty-eight subjects with asthma and 22 normal controls were included in the study. Patients were recruited from Glenfield hospital in Leicester, the United Kingdom. Subjects were characterized using clinical history and lung function. Asthma was diagnosed as described previously [33]. Asthmatic subjects had a suggestive history with recurrent cough, wheeze, sputum production and either airway hyperresponsiveness [Provocatioanal concentration that causes a 20% fall in FEV_1_ (Pc20)<8 mg/mL] or reversible airflow obstruction [forced expiratory volume in 1 s (FEV_1_)>12%]. Normal subjects had no history of respiratory disease. Methacholine challenge was performed using a Wright's nebulizer and results are expressed as the inducing concentration of methacholine required to elicit a 20% decrease in FEV_1_ (Pc20). Atopic status was assessed by measurement of the total IgE concentration and skin prick tests to common aeroallergens (a weal diameter >3 mm compared with the control was defined as positive). The clinical characteristics are given in Table 1. Not all subjects had a bronchoscopy. The clinical characteristics of the subset of those subjects who had a bronchoscopy are given separately with Table 1.

**Table 1:** The characteristics of all asthmatics and normal subjects who were studied are given in the first two columns

	Normal	Asthma	Bronchoscopy normal	Bronchoscopy asthma
*N*	22	38	7	13
Age*	29 (19–56)	43 (21–61)	24 (21–49)	46 (36–60)
Male	8	21	3	5
Atopy	6	19	1	4
IgE† (kUa/L)	17 (7.2)	525 (143)	101 (34)	277 (117)
FEV_1_% Pred†	89 (5.4)	85 (3.3)	100 (6.3)	77 (5.9)
Pc20‡	>16	0.5 (0.8)	>16	0.9 (0.7)
On OCS	0	8	0	5

* Median (range).

† Mean (standard error of mean).

‡ Geometric mean (log SD).

The characteristics of only those who underwent bronchoscopy are given separately in the last two columns.

All except six asthmatics (mild intermittent asthma) were using regular inhaled corticosteroids. Eight asthma subjects were using oral as well as inhaled corticosteroids. Twenty out of the 38 asthmatics had severe asthma according to the British Thoracic Society criteria (steps 4 and 5). Bronchoscopy was performed on 13 of the asthmatics and seven normal subjects. Of the 13 asthmatics investigated with bronchoscopy, 10 had severe asthma and of these five were on oral corticosteroids (OCS). The study was approved by the Leicestershire ethics committee and written informed consent was obtained from all participants.

### Bronchoscopy

Ten millilitre of venous blood was obtained before the procedure for isolation of peripheral blood mononuclear cells (PBMC). Bronchoscopy was performed using local anaesthesia according to standard techniques. Asthmatic subjects were pre-medicated with 2.5 mg of nebulized Salbutamol 20 min before the procedure. Mild sedation with midazolam was used in some subjects. BAL was performed by sequential instillation and aspirations of three aliquots of 60 mL of warmed sterile 0.9% saline. BAL fluid was placed and transported in ice before analysis. Biopsies were first fixed in 4% paraformaldehyde and then embedded in paraffin.

### Isolation of peripheral blood mononuclear cells and bronchoalveolar lavage T cells

Ten to fifteen millilitre of heparinized venous blood was obtained from all the subjects. PBMC was isolated from whole blood using density gradient centrifugation (Histopaque 1077; Sigma, Dorset, UK). PBMC were washed and suspended in phosphate-buffered saline (PBS) (Invitrogen, Paisley, UK)+0.5% bovine serum albumin (BSA) (Sigma) during staining and subsequent flow cytometry. BAL samples were first filtered through 48 μm nylon gauze to remove debris. Cells were pelleted and a red cell lysis was performed if significant blood contamination had occurred. Supernatants were collected and stored at −80 °C for estimation of CCL1. Red cell lysis was performed by incubating the cellular pellet with a small volume of ice-cold distilled water for 20 s, followed by quenching with a large volume of isotonic PBS.

### Labelling CCR8

Either anti-CCR8 mAb (433H) or the fluorescent-labelled CCL1 (CCL1-F) was used to identify CCR8^+^ cells. The 433H is an unconjugated mouse IgG2a mAb directed against human CCR8, and was a gift from ICOS Corporation (Seattle, WA, USA). The anti-CCR8 antibody was generated by injecting mice three times intra-peritoneally with 5 × 10^6^ COS cells, which were transfected to express high levels of CCR8 (shown by a chemotactic response to CCL1), suspended in an RIBI MPL+TDM adjuvant (Corixa/GSK Biologicals, Hamilton, MT, USA). Hybridoma fusion well antibody supernatants were screened by FACS on the CCR8 transfectants, using non-transfected cells as the negative control. Supernatants were also tested for the ability to block cell chemotaxis towards CCL1. Positive wells were cloned, resulting in the identification of monoclonal antibody 433H. Purified 433H mAb was shown to be specific for CCR8 by FACS and in chemotaxis inhibition assays using a panel of cell lines transfected with a comprehensive panel of chemokine receptors (data not shown). The Alexa Fluor 647-labelled CCL1 (CCL1-F) was a kind gift provided by Millennium Pharmaceuticals. The Alexa Fluor 647 signal was detected in the FL3 channel during acquisition on the flow cytometer.

### Flow cytometry

This was carried out as described previously [22]. All samples were labelled with anti-CCR8 or CCL1-F and CD3. Anti-CD4, anti-CD8 (BD Biosciences, Oxford, UK) and anti-CD45RO (Caltag, Buckingham, UK) were also used in some samples. PBMC and BAL cells were incubated with anti-CCR8 mAb at 4 °C while suspended in PBS and 0.5% BSA. FITC or Phycoerythrin-labelled anti-mouse, rabbit antibodies (Dako Cytomation, Cambridge, UK) were used as the secondary antibody. Cells were suspended in MACS buffer (Ca^2+^/Mg^2+^ Dulbecco's phosphate-buffered saline and 0.5% BSA and 5 mm EDTA) for labelling with CCL1-F and this was carried out at 37 °C. Intracellular cytokine was studied in some of the samples. Where intracellular cytokine was studied, cells were first incubated with anti-CCR8 or CCL1-F and then stimulated using 25 ng/mL of Phorbol–Myristate–Acetate (Sigma) and 500 ng/mL of Calcium Ionophore A2317 (Sigma) in the presence of 10 μg/mL Brefeldin (Sigma) for 6 h at 37 °C. Cells were then washed and fixed using 4% Paraformaldehyde before being permeabilized with a 0.1% solution of Saponin. Cells were then stained for intracellular IL-4, IL-13 and IFN-γ using directly labelled mAbs, followed by CD3-PerCP. Samples were analysed on FACS Canto using FACS Diva software (Becton Dickinson Immunocytometry system). At least 10 000 live cells were acquired from each sample for the analysis of the surface chemokine receptor. The percentage of CD3^+^ cells that were CCR8^+^ was estimated. The percentages of CD3^+^ cells that were cytokine positive were low and on average 30 000–40 000 PBMC and BAL cells were analysed from each sample when intracellular cytokines were studied. Isotype controls were included for all tests.

### CCL1 quantification

CCL1 in BAL fluid was assayed using a commercially available ELISA kit (R&D Systems Inc., Minneapolis, MN, USA) with a detection limit of 15 pg/mL. We had not been able to detect any CCL1 in unconcentrated BAL samples from asthmatic or normal subjects. BAL samples were therefore 20-fold concentrated using Amicon Ultra-15 centrifugal filters (for nominal molecular weight limit 5000) (Millipore, Watford, UK). BAL samples were centrifuged within the centrifugation tubes at 400 **g** for 30 min at 25 °C. The filtrate volumes recovered were 0.25–1.0 mL. Samples were assayed by ELISA in duplicate and the product protocol was followed. CCL1 values <15 pg/mL were considered undetectable.

### T cell polarization

T cell polarization was performed using a method adapted from Cousins et al. [34]. *In vitro* polarized IFN-γ^+^ and IL-4^+^ T cells were expanded from PBMC isolated from eight asthmatic subjects. CD3 cells were stimulated to proliferate, using anti-CD3/CD28 beads (Dynabeads® CD3/CD28 T cell expander; Invitrogen, UK), in the presence of recombinant human IL-2. To direct *in vitro* polarization towards IFN-γ-producing T cells, rIL-12 (2.5 ng/mL; R&D Systems) and anti-IL-4 (0.1 μg/mL PreproTech EC Ltd, London, UK) were added to the culture well. To direct *in vitro* polarization towards IL-4-producing T cells, rhIL-4 (12.5 ng/mL purchased from R&D Systems) and anti-IFN-γ antibodies (0.1 μg/mL PeproTech EC Ltd, UK) were added to the culture.

### Chemotaxis assay

Chemotaxis assay was performed in a modified Boyden chamber assay using *in vitro* polarized IL-4^+^ T cells with an upregulated expression of CCR8 (19–27% vs. ∼5% in fresh PBMC). Migration towards recombinant human CCL1 (R&D Systems) was assessed at a concentration of 100 ng/mL diluted in RPMI 1640+10% FCS. CCL1 was placed in the lower wells of a Transwell® chemotaxis plate (Sigma-Aldrich, Dorset, UK). A 100 μL suspension of polarized cells at a concentration of 10 × 10^6^/mL cells was added to the 3 μm pore size polycarbonate culture insert. Cells pre-incubated with CCR8 mAb or the isotype IgG_2A_ were also included. SDF-1 (CXCL12) was used as the positive control and a lower well without rhCCL1 was used as the negative control. The plate was incubated at 37 °C for 2 h in a humidified 5% CO_2_ incubator. Migrated cells in the lower wells were recovered and counted at magnification × 20. Migrating cells are expressed as a percentage of cells initially added to the culture insert. Assays were performed in duplicate.

### Immunohistochemistry and cytospins

Biopsies were fixed in 4% paraformaldehyde overnight and then embedded in paraffin blocks. These were then cut to obtain 4-μm-thick sections. Antigen retrieval was performed by immersing sections in a pH 6.0, 100 mm citrate buffer (Lab Vision, Cheshire, UK) and heating to 120 °C in a pressurized container (PASCAL, Dako). CCR8 mAb and isotype control antibody was used at a concentration of 10 μg/mL. Biotinylated anti-mouse/anti-rabbit IgG (H+L) was used as the secondary antibody. The Streptavidin ABC complex was used as the detection system. Sub-epithelium was identified morphologically. Positive cells within the area of the sub-epithelium were enumerated at a magnification of × 400 and expressed as number of cells per mm^2^. The area covered by smooth muscle was excluded. A minimum of 0.1 mm^2^ was analysed in each biopsy section. Image analysis software (Scion Image) was used to estimate the surface area.

### Statistical analysis

Subject characteristics are described using descriptive statistics. Means are expressed together with standard error of mean (SEM). Median values are expressed with interquartile ranges (IQR). Statistical analysis was performed with GraphPad Prism (GraphPad software Inc.). Differences between groups were assessed using the Mann–Whitney *U*-test for non-parametric and unpaired *t*-tests for parametric data. Pearson's correlation test was used to assess the relationship between receptor expression and clinical parameters. A *P* value <0.05 was considered statistically significant. This was an explorative study and power calculation was not possible as the level and variation of the expression of CCR8 are not known. Sufficient numbers of subjects were included for the experiments on blood. The number of subjects used for experiments with BAL were small; nevertheless, we did detect a difference between the two populations compared.

## Results

### CCR8 expression was twofold increased on *in vitro* polarized IL-4^+^ T cells compared with IFN-γ^+^ T cells in asthma

**Fig. 1:**
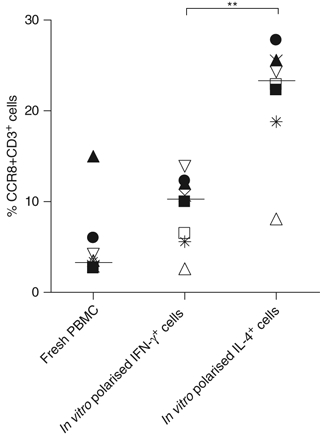
T cell CCR8 expression among fresh peripheral blood mononuclear cells (PBMC), *in vitro* polarized IFN-γ^+^ and IL-4^+^ cells. Graph shows data from eight different experiments using PBMC from eight different asthmatic subjects. Bars represent median percentages. (^**^P<0.01 using the Mann–Whitney test).

We studied the effect of *in vitro* polarization to IL-4^+^ and IFN-γ^+^ T cells on the expression of CCR8. This allowed us to see both whether CCR8 was preferentially associated with IL-4^+^ T cells and to test the selectivity of the new anti-CCR8 mAb against the human CCR8 receptor in chemotaxis assays (described below). IL-4^+^ and IFN-γ^+^ T cells were expanded *in vitro* over 2 weeks from PBMC derived from eight asthmatic donors. The mean CCR8 expression on T cells from these donors before polarization was 5.3±1.6% (Fig. 1). Expression was significantly increased on IL-4^+^ polarized T cells (21.9±2.1%) compared with IFN-γ^+^ polarized T cells (9.2±1.3) (*P*<0.01).

**Fig. 2:**
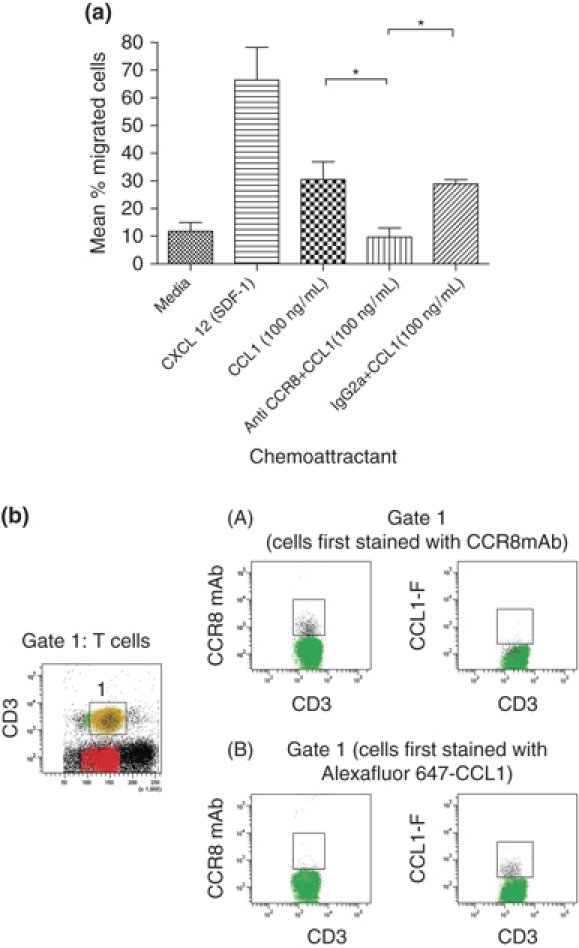
(a) Chemotaxis of *in vitro* polarized IL-4^+^ cells towards rhCCL1. SDF-1 was used as the positive control. Migration towards CCL1 was blocked by pre-incubating cells with anti-CCR8 mAb (433H). Graph shows data from six different experiments. Columns represent mean percentages and error bars standard error of mean. ^*^P<0.05 using the Mann–Whitney test. (b) Representative FACS plot showing the binding of anti-CCR8 mAb being blocked by Alexafluor 647-labelled CCL1 (CCL1-F). (A) Cells are incubated with CCR8 mAb (433H) and then CCL1-F. (B) Cells are incubated with CCL1-F first and then CCR8 mAb.

Previous reports of CCR8 in human asthma had been difficult to interpret due to the poor specificity of the anti-CCR8 antibody used. We have confirmed the specificity of the 433H anti-CCR8 mAb using two methods: chemotaxis experiments and blocking experiments using the novel fluorescence-labelled-CCL1 (CCL1-F). Chemotaxis experiments were performed using IL-4^+^ polarized T cells. The mean CCR8 expression on these cells was 21.9±2.1%. Approximately 30% of the IL-4^+^ polarized T cells migrated towards CCL1 compared with 10% in the control well (Fig. 2a). This response was completely abrogated by pre-incubation of cells with the anti-CCR8 (433H) mAb but not the isotype control antibody. In blocking experiments, we have pre-incubated PBMC with CCL1-F before labelling with anti-CCR8 mAb. The CCL1-F completely blocked the staining by anti-CCR8 (433H). Similarly, pre-incubating PBMC with the anti-CCR8 antibody completely blocked the binding of CCL1-F to the CCR8 receptor (Fig. 2b).

### There were more CCR8^+^ T cells in the blood from subjects with asthma compared with normal controls

**Fig. 3:**
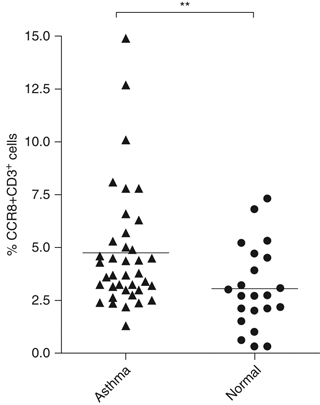
Scatter plot of CCR8 expression on blood T cells in asthmatics (▴) and normal subjects (•). ^**^P<0.01 using the Mann–Whitney test. Bars represent the mean percentages.

CCR8 expression among blood T cells was confined to a small percentage of T cells in both asthmatic and normal subjects (normal controls 3.0±0.4% and asthmatics 4.7±0.4%). The percentage of CCR8^+^ T cells was significantly higher in asthmatic subjects compared with normal controls (*P*<0.01) (Fig. 3). CCR8 expression was mostly confined to CD3^+^ cells among the PBMC population. The majority of CCR8^+^ T cells were CD45R0^+^ (77±4.3%; *n*=3) and CD4^+^ T cells (82.3±2.8; *n*=6). 15±2.1% of CD8^+^ T cells expressed CCR8 (*n*=6). We had also explored whether there was any association of CCR8 expression with serum markers of allergy (IgE and eosinophils) in both asthmatics and normal controls. There was no significant association between the percentage of CCR8^+^ T cells and serum IgE (*r*^2^=0.14; *P*=0.4) or blood eosinophil count (*r*^2^=0.267; *P*=0.09).

### CCR8 expression was enriched on IL-4^+^/IL-13^+^ T cells compared with IFN-γ^+^ T cells in blood from asthma and normal subjects

**Fig. 4:**
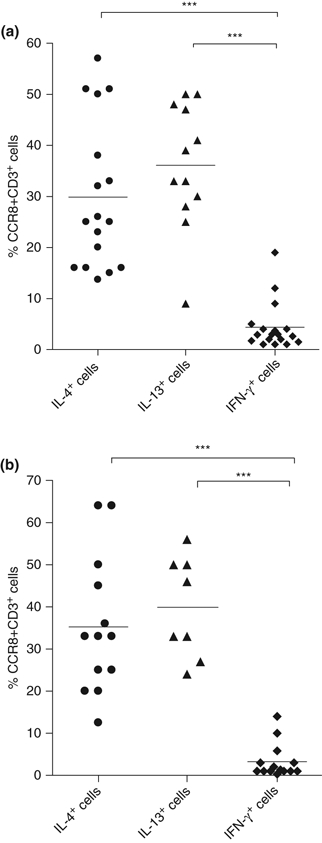
(a) Scatter plot of CCR8 expression on IFN-γ^+^ (♦), IL-4^+^ (•) and IL-13^+^ (▴) peripheral blood mononuclear cells (PBMC) derived CD3^+^ cells from asthmatic subjects. Cells were incubated with PMA and calcium ionophore for 6 h before staining with anti-IFN-γ, anti-IL-4 or anti-IL-13. ^***^P<0.001 using the Mann–Whitney test. Bars represent mean percentages. (b) Scatter plot of CCR8 expression on IFN-γ^+^ (♦), IL-4^+^ (•) and IL-13^+^ (▴) PBMC-derived CD3^+^ cells from normal subjects. Cells were incubated with PMA and calcium ionophore for 6 h before staining with anti-IFN-γ, anti-IL-4 or anti-IL-13. ^***^P<0.001 using the Mann–Whitney test. Bars represent the mean percentages.

We studied the association of CCR8 with IL-4^+^ T cells by analysing their expression on IFN-γ^+^, IL-4^+^ and IL-13^+^ T cells following *in vitro* stimulation with PMA and calcium ionophore. We confirmed that stimulation did not affect CCR8 expression (data not shown). In subjects with asthma, 29.8±3.5% and 36±3.5% of the IL-4^+^ and IL-13^+^ blood T cells expressed CCR8 while only 4.3±1.0% of IFN-γ^+^ cells expressed CCR8 (*P*<0.001) (Fig. 4a). A similar degree of enrichment of CCR8^+^ cells on IL-4^+^ and IL-13^+^ T cells relative to IFN-γ^+^ T cells was seen in the blood from normal subjects (Fig. 4b).

### There were more CCR8^+^ T cells in the bronchoalveolar lavage of asthmatics compared with normal subjects

**Fig. 5:**
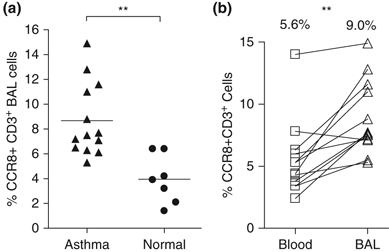
(a) Scatter plot of CCR8^+^ CD3^+^ cells in bronchoalveolar lavage (BAL) samples from asthmatic (▴) and normal subjects (•). ^**^P<0.01 using the Mann–Whitney test. Bars represent mean percentages. (b) CCR8^+^ CD3^+^ cells in matched BAL (Δ) and blood (□) samples from the same subject in asthmatics. ^**^P<0.01 using the Mann–Whitney test. Lines connect samples from the same subject. Mean percentages are given at the top of the figure.

Previous flow cytometry-based studies of CCR in BAL have shown higher percentages of CCR4, CCR2, CCR6 and CXCR6 in the lung compared with blood CD3^+^ cells; however, none have shown a difference between asthmatic and normal controls. Studies of CCR8 expression on BAL cells have been limited by the lack of availability of a reliable antibody. We studied BAL samples from 13 asthmatics and seven normal subjects for CCR8 expression. There were significantly more CCR8-expressing T cells in BAL from asthmatics compared with normal controls (mean percentage 8.6±0.8% and 3.9±0.7%, respectively) (*P*<0.01) (Fig. 5a). There was also a significant increase in the percentage of CCR8^+^ T cells in BAL compared with blood within the same subject in asthmatics (9.03±0.9% vs. 5.69±0.94%) (*P*<0.01). This was not observed in normal subjects (Figs 5a and b).

### CCR8 is preferentially expressed on IL-4^+^ T cells in bronchoalveolar lavage

**Fig. 6:**
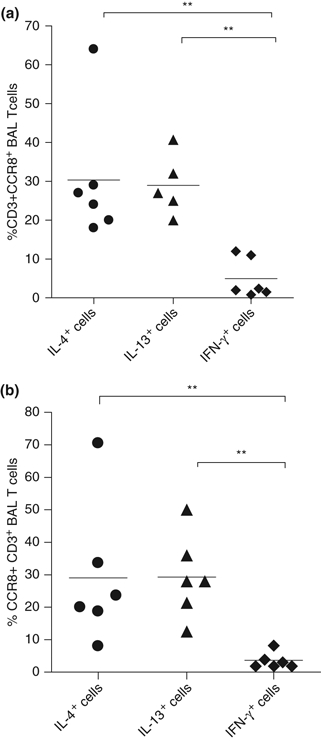
Scatter plot of CCR8 expression on IFN-γ^+^ (♦), IL-4^+^ (•) and IL-13^+^ (▴) BAL CD3^+^ cells from (a) asthma subjects and (b) normal subjects. Cells were first stimulated with PMA and calcium ionophore. ^**^P<0.01 using the Mann–Whitney test. Bars represent mean.

As with blood T cells, CCR8 was enriched on IL-4^+^ T cells in BAL. In asthma, CCR8 was expressed by 30.3±3.0% and 28.9±7.8% of the IL-4^+^- and IL-13^+^-producing BAL CD3^+^ cells, respectively. In contrast, only 4.9±2.0% of IFN-γ^+^ BAL CD3^+^ cells expressed CCR8 (Fig. 6a). A similar pattern of CCR8 expression was seen on IL-4^+^- and IL-13^+^-producing BAL T cells from normal subjects (Fig. 6b).

### There was a higher concentration of CCL1 in bronchoalveolar lavage of asthmatics compared with normal controls

**Fig. 7:**
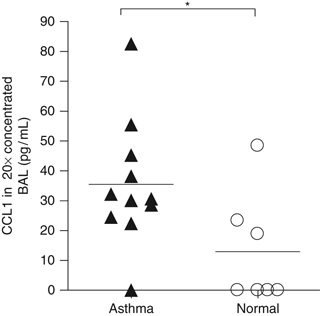
Concentration of CCL1 in 20-fold concentrated BAL fluid from asthmatic (▴) and normal subjects (○). ^*^P<0.05 using the Mann–Whitney test. Bars represent the mean concentrations.

We found a higher percentage of CCR8^+^ T cells in the BAL of asthmatic subjects compared with normal controls. We investigated whether the ligand for CCR8 (CCL1) was present in increased concentrations in the BAL fluid of asthmatics compared with normal controls. Preliminary studies showed that CCL1 was not detectable in BAL fluid but was detectable following concentration. We used ultracentrifugation filters to concentrate BAL fluid samples 20-fold. The mean concentration of CCL1 in 20 × BAL fluid from asthmatic subjects was significantly higher than in that in normal subjects (35±6 vs. 12.9±7 pg/mL; *P*<0.05) (Fig. 7).

### There were more CCR8-positive cells in bronchial biopsies from asthmatic compared with normal subjects

**Fig. 8:**
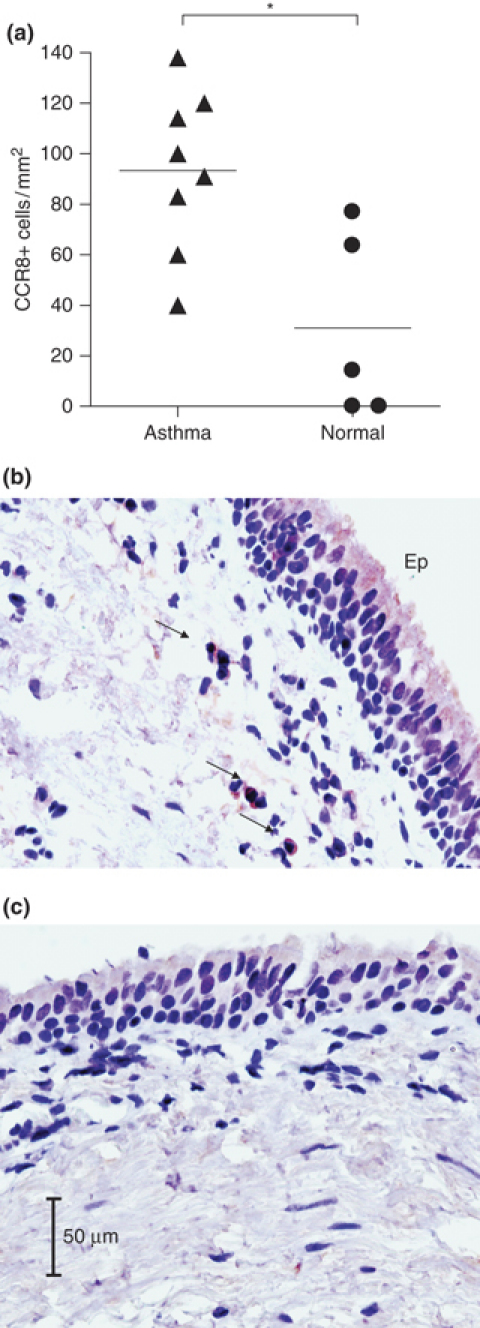
(a) CCR8^+^ cells/mm^2^ within the bronchial mucosa of asthma (*n*=8) (▴) and normal control subjects (*n*=5) (•). ^*^P<0.05 using the Mann–Whitney test. Bars represent the mean CCR8^+^ cells/mm^2^. (b) Bronchial biopsy section from an asthmatic subject stained with anti-CCR8 mAb (433H) (black arrows indicate CCR8^+^ cells) and (c) IgG2a isotype control. Ep, Epithelium. Scale bar 50 μm.

In addition to blood and BAL fluid, we also investigated the presence of CCR8^+^ T cells within the bronchial mucosa of asthmatic and normal subjects. Biopsies were obtained from five normal and eight severe asthmatics. The 433H anti-CCR8 mAb only showed staining in paraffin-embedded specimens. The thickness of sections produced using paraffin-embedded specimens did not allow accurate co-localization of CD3^+^ and CCR8^+^ cells. Most, if not all, blood CCR8^+^ were CD3^+^ on flow cytometry studies, and so those cells that were CCR8^+^ are probably all T cells. The mean±SEM of CCR8^+^ cells seen within the sub-epithelium of the bronchial biopsies taken from asthmatics was 93±11 cells/mm^2^ compared with 31±16 cells/mm^2^ in normal subjects (*P*<0.05) (Figs 8a–c). CCR8^+^ cells were seen mostly intra-epithelium and in the sub-epithelium.

## Discussion

In this study, we have shown for the first time that there are increased numbers of CCR8^+^ T cells in the BAL of asthmatics compared with normal subjects. Moreover, in paired blood and BAL samples from asthmatics, there were more CCR8-expressing T cells in BAL than in blood. This difference could be due to the recruitment of CCR8-expressing T cells from blood to the lung, a possibility supported by the increased concentration of CCL1 in BAL fluid from asthmatics compared with normal subjects. This study therefore provides further evidence for a potential role for CCR8 in lung T cell recruitment in clinical asthma.

The investigation of the role of CCR8 in human disease has been hampered by the lack of robust reagents to detect CCR8 expression on the cell surface. We have for example been unable to obtain reliable staining with the two commercially available anti-CCR8 antibodies (data not shown). For this study, we had access to a well-characterized, specific, blocking, monoclonal antibody from ICOS Corporation, which could be used in both flow cytometry and immunohistochemistry. We also found that this antibody showed good specificity for the CCR8 receptor. In chemotaxis assays using *in vitro* polarized IL-4^+^ cells, we found that the antibody effectively inhibited CCL1-mediated T cell migration. In addition, CCL1-F yielded the same amount of staining for CCR8 as the anti-CCR8 antibody and pre-incubation with the labelled CCL1 blocked subsequent binding of the anti-CCR8 antibody to T cells. Similarly, the anti-CCR8 antibody also effectively blocked binding of the CCL1-F to CCR8. We are therefore confident that this antibody recognizes human CCR8.

Our data support the findings from human *in vitro* studies of CCR8 expression by T_H_2 cells. After 2 weeks of *in vitro* culture under T_H_2 polarizing conditions, the percentage of CD3^+^ cells expressing CCR8 was significantly increased compared with that cultured under T_H_1 polarizing conditions. We have also demonstrated the association of CCR8 expression with IL-4^+^ T cells *ex vivo* using blood and BAL T cells by simultaneous intracellular cytokine and surface CCR8 measurement. There was an approximately sixfold enrichment of CCR8 expression on IL-4^+^ T cells compared with IFN-γ^+^ T cells. We acknowledge that the percentages of IL-4^+^/IL-13^+^ T cells in our study also included CD8^+^ T cells (Tc2). Furthermore, cells in our study were single stained with anti-IL-4 or anti-IFN-γ, and some of the IL-4^+^ T cells may also be IFN-γ^+^ (T_H_0 cells). This would also apply to the *in vitro* polarized T cells. Given these limitations, we would not be able to suggest a definite association of CCR8 with T_H_2 cells using our data. Nevertheless, CCR8 expression was seen on a significantly higher percentage of IL-4^+^ and IL-13^+^ T cells compared with IFN-γ^+^ T cells among blood and BAL T cells in both asthmatics and normal subjects.

Our findings on the expression of CCR8 on peripheral blood cells were similar to the recently reported findings of Soler et al. [24], who used CCL1-F to identify CCR8-expressing cells in human peripheral blood. They noted a predominant expression on CD4^+^CD45RO^+^ T cells, with an approximately sixfold enhancement of expression on T_H_2-cytokine producing T cells. They also found increased expression of CCR8 on FOXP3-expressing T_reg_ cells, accounting for ∼60% of this group of T_reg_ cells. Among CCR8^+^ T cells, ∼20% were shown to express FOXP3. In this study, we had noted a significantly higher percentage of CCR8 expression on peripheral blood T cells from asthmatics, which has not been observed for any other chemokine receptors. There was no statistical difference between the expression of CCR8 among PBMC from severe compared with mild-moderate asthmatics (mean percentages of 5.2 vs. 4.1).

Our most striking finding was the greater percentage of BAL T cells expressing CCR8 in asthmatics compared with normal controls. Previous studies have demonstrated higher percentages of CCR2, CCR4, CCR5, CCR6, CXCR3 and CXCR6 among lung T cells compared with blood. However, there was no difference in any of the T cell-expressed chemokine receptors between asthmatics and normal subjects including the T_H_2 cell-associated receptors CCR3 and CCR4, and so what we have observed appears to be specific for CCR8 [18, 22, 35, 36]. This may be because previous studies of chemokine receptor expression in asthma have been on mild steroid naïve asthmatics, whereas our subjects, who underwent bronchoscopy, had more severe disease. Unlike our previous studies, we did find a significant although modest twofold increase in the percentage of IL-4/IL13-expressing BAL T cells in the asthmatics compared with the healthy subjects (data not shown), which would be consistent with the approximately twofold increase in the percentage of CCR8-expressing cells. It is always possible that the increased expression of CCR8 compared with normal subjects was due to treatment, in particular steroids. However, as we found no difference in the expression of CCR8 in blood or BAL in patients taking oral steroids vs. those only on inhaled steroids, we feel this is unlikely.

It is not possible to say whether the increased CCR8 expression was due to the activation of BAL T cells or increased recruitment of CCR8-expressing T cells from the blood. However, in support of the latter mechanism, we did observe an increase in the CCL1 concentrations in BAL fluid. This was at low concentrations, well below the amount required for chemotaxis. However, it is difficult to extrapolate from concentration in BAL the site of release and action in the lung. One other group has also reported increased CCL1 in BAL fluid in asthmatics, although another group was not able to detect CCL1 in allergen-challenged asthmatics [31, 32]. CCL1 was not detected in bronchial biopsies following an allergen challenge in one study [21]. However, increased expression of CCL-1 was observed by *in situ* hybridization by another group [28]. There have also been reports of CCL-17 (found at higher levels in asthma BAL compared with normal controls) being a ligand for CCR8, although this observation was not supported by another group [37, 38]. The extent to which there is a chemotactic signal for CCR8^+^ cells in asthmatic airways therefore needs further investigation.

Three reports have been published of CCR8 expression in the airways in asthma, all studying bronchial biopsies [21, 28, 30]. In the first of these reports, CCR8 expression was studied using a goat polyclonal antibody raised against an extracellular peptide in bronchial biopsies after allergen challenge [21]. In excess of 250 CCR8^+^ T cells/mm^2^ were detected at baseline and a mean of 1000 after allergen challenge. In contrast, a second study, also using a goat polyclonal antibody from the same company, found just 4 cells/mm^2^ staining positive for CCR8 in the mucosa of moderate to severe asthmatics and no difference from controls [30].

A third study studied CCR8 mRNA in bronchial biopsies using *in situ* hybridization [28]. Ten asthmatics were compared with seven normal controls. Increased CCR8 mRNA was shown in the asthmatic biopsies compared with normal controls (27.3 vs. 7.4 cells/mm^2^), the majority of which were CD4 T cells. Our study supports the findings of this study, although we found higher numbers of positive cells in the mucosa. The study by Gonzalo and colleagues had also shown increased staining for CCL1 in the bronchial biopsies of asthmatics. Interestingly, they had also shown that CCL1 was the major chemokine secreted by mast cell lines following FcɛR1 activation. A mast cell-driven recruitment of T_H_2 cells to the asthmatic airway was proposed by Gonzalo et al. [28].

The exact contribution made by CCR8-expressing T cells would need further detailed study, especially so with the development of CCR8 antagonists that may have potential clinical use [39]. Current evidence suggests that CCR8-expressing T cells may have multiple roles. It is possible that a significant proportion of CCR8-expressing cells are T_regs_ and CCR8 may play a role in the trafficking of these cells [24, 40]. However, given their inhibitory effect on immune processes including T_H_2 cells [41], one would expect their numbers to be decreased in asthma. On the other hand, the percentage of T_reg_ cells in blood is known to be increased with the use of inhaled and oral glucocorticoids [42]. All patients who had participated in the bronchoscopic part of the study were taking high doses of inhaled steroids and some were also taking OCS.

CCR8 may act in concert with other chemokine receptors in guiding certain T cell subsets to tissue sites. CCR8^+^ cells that also expressed CLA were shown to have equal frequencies of T_H_1 and T_H_2 cells, whereas T_H_2 cells were several folds enriched in the CLA^−^ CCR8^+^ population, suggesting that CCR8 may be one of a combination of receptors guiding T_H_2 cells to disease sites [24]. Our study provides evidence that CCR8 may be involved in human asthma and may contribute to its pathogenesis in part through its role in trafficking T_H_2 cells to the lung. CCR8 may potentially be an important target for therapeutic intervention.

## References

[1] Wardlaw AJ, Brightling CE, Green R, Woltmann G, Bradding P, Pavord ID (2002). New insights into the relationship between airway inflammation and asthma. Clin Sci (London).

[2] Gavett SH, Chen X, Finkelman F, Wills-Karp M (1994). Depletion of murine CD4+T lymphocytes prevents antigen induced airway hyperreactivity and pulmonary eosinophilia. Am J Respir Cell Mol Biol.

[3] Azzawi M, Bradley B, Jeffrey P (1990). Identification of activated T lymphocytes and eosinophils in bronchial biopsies in stable atopic asthma. Am Rev Respir Dis.

[4] Bentley AM, Menz G, Storz C (1992). Identification of T lymphocytes, macrophages and activated eosinophils in the bronchial mucosa in intrinsic asthma. Am Rev Respir Dis.

[5] Larché M, Robinson DS, Kay AB (2003). The role of T lymphocytes in the pathogenesis of asthma. J Allergy Clin Immunol.

[6] Wills-Karp M (1999). Immunological basis of antigen-induced airway hyperresponsiveness. Ann Rev Immunol.

[7] Romagnani S (2001). T-cell responses in allergy and asthma. Curr Opin Allergy Clin Immunol.

[8] Robinson DS (2000). Th-2 cytokines in allergic disease. Br Med Bull.

[9] Robinson DS, Hamid Q, Ying S (1992). Predominant TH2-like bronchoalveolar T-lymphocyte population in atopic asthma. N Engl J Med.

[10] Nakamura Y, Ghaffar O, Olivenstein R (1999). Gene expression of the GATA-3 transcription factor is increased in atopic asthma. J Allergy Clin Immunol.

[11] Humbert M, Menz G, Ying S (1999). The immunopathology of extrinsic (atopic) and intrinsic (non-atopic) asthma: more similarities than differences. Immunol Today.

[12] Borgonovo B, Casorati G, Frittoli E, Gaffi D, Crimi E, Burastero SE (1997). Recruitment of circulating allergen-specific T lymphocytes to the lung on allergen challenge in asthma. J Allergy Clin Immunol.

[13] Gerblich AA, Campbell AE, Schuyler MR (1984). Changes in T-lymphocyte subpopulations after antigenic bronchial provocation in asthmatics. N Engl J Med.

[14] Rossi D, Zlotnik A (2000). The biology of chemokines and their receptors. Ann Rev Immunol.

[15] Mackay C (2001). Chemokines: immunology's high impact factors. Nat Immunology.

[16] Campbell JJ, O'Connell DJ, Wurbel MA (2007). Cutting edge: chemokine receptor CCR4 is necessary for antigen-driven cutaneous accumulation of CD4 T cells under physiological conditions. J Immunol.

[17] Kunkel EJ, Cambell JJ, Haraldson G (2000). Lymphocyte CC chemokine receptor 9 and epithelial thymus-expressed chemokine (TECK) expression distinguish the small intestine immune compartment: epithelial expression of tissue-specific chemokines as an organising principle in regional immunity. J Exp Med.

[18] Campbell JJ, Brightling CE, Symon FA (2001). Expression of chemokine receptors by lung T cells from normal and asthmatic subjects. J Immunol.

[19] Panina-Bordignon P, D'Ambrosio D (2003). Chemokines and their receptors in asthma and chronic obstructive pulmonary disease. Curr Opin Pulm Med.

[20] Wenzel SE, Trudeau JB, Barnes S (2002). TGF-beta and IL-13 synergistically increase eotaxin-1 production in human airway fibroblasts. J Immunol.

[21] Panina-Bordignon P, Papi A, Mariani M (2001). The C–C chemokine receptors CCR4 and CCR8 identify airway T cells of allergen-challenged atopic asthmatics. J Clin Invest.

[22] Morgan AJ, Symon FA, Berry MA, Pavord ID, Corrigan CJ, Wardlaw AJ (2005). IL-4-expressing bronchoalveolar T cells from asthmatic and healthy subjects preferentially express CCR 3 and CCR 4. J Allergy Clin Immunol.

[23] Zingoni A, Soto H, Hedrick JA (1998). The chemokine receptor CCR8 is preferentially expressed in Th2 but not Th1 cells. J Immunol.

[24] Soler D, Chapman TR, Poisson LR (2006). CCR8 expression identifies CD4 memory T cells enriched for FOXP3+regulatory and Th2 effector lymphocytes. J Immunol.

[25] Goya I, Villares R, Zaballos A (2003). Absence of CCR8 does not impair the response to ovalbumin-induced allergic airway disease. J Immunol.

[26] Chung CD, Kuo F, Kumer J (2003). CCR8 is not essential for the development of inflammation in a mouse model of allergic airway disease. J Immunol.

[27] Mikhak Z, Fukui M, Farsidjani A, Medoff BD, Tager AM, Luster AD (2009). Contribution of CCR4 and CCR8 to antigen-specific T(H)2 cell trafficking in allergic pulmonary inflammation. J Allergy Clin Immunol.

[28] Gonzalo JA, Qiu Y, Lora JM (2007). Coordinated involvement of mast cells and T cells in allergic mucosal inflammation: critical role of the CC chemokine ligand 1:CCR8 axis. J Immunol.

[29] Buckland KF, O'Connor EC, Coleman EM, Lira SA, Lukacs NW, Hogaboam CM (2007). Remission of chronic fungal asthma in the absence of CCR8. J Allergy Clin Immunol.

[30] Ying S, O'Connor B, Ratoff J (2008). Expression and cellular provenance of thymic stromal lymphopoietin and chemokines in patients with severe asthma and chronic obstructive pulmonary disease. J Immunol.

[31] Montes-Vizuet R, Vega-Miranda A, Valencia-Maqueda E, Negrete-Garcia MC, Velasquez JR, Teran LM (2006). CC chemokine ligand 1 is released into the airways of atopic asthmatics. Eur Respir J.

[32] Bochner BS, Hudson SA, Xiao HQ, Liu MC (2003). Release of both CCR4-active and CXCR3-active chemokines during human allergic pulmonary late-phase reactions. J Allergy Clin Immunol.

[33] Brightling CE, Symon FA, Birring SS, Bradding P, Pavord ID, Wardlaw AJ (2002). Th2 cytokine expression in bronchoalveolar lavage T-lymphocytes and bronchial submucosa is a feature of asthma and eosinophilic bronchitis. J Allergy Clin Immunol.

[34] Cousins D, Lee TH, Staynov D (2002). Cytokine expression during human Th1/Th2 cell differentiation: direct evidence for coordinated expression of Th2 cytokines. J Immunol.

[35] Morgan AJ, Guillen C, Symon FA (2005). Expression of CXCR6 and its ligand CXCL16 in the lung in health and disease. Clin Exp Allergy.

[36] Kallinich T, Schmidt S, Hamelmann E (2005). Chemokine-receptor expression on T cells in lung compartments of challenged asthmatic patients. Clin Exp Allergy.

[37] Bernardini G, Hedrick J, Sozzani S (1998). Identification of the CC chemokines TARC and macrophage inflammatory protein-1 beta as novel functional ligands for the CCR8 receptor. Eur J Immunol.

[38] Garlisi CG, Xiao H, Tian F (1999). The assignment of chemokine-chemokine receptor pairs: TARC and MIP-1 beta are not ligands for human CC-chemokine receptor 8. Eur J Immunol.

[39] Ghosh S, Elder A, Guo J (2006). Design, synthesis, and progress toward optimization of potent small molecule antagonists of CC chemokine receptor 8 (CCR8). J Med Chem.

[40] Iellem A, Mariani M, Lang R (2001). Unique chemotactic response profile and specific expression of chemokine receptors CCR4 and CCR8 by CD4(+)CD25(+) regulatory T cells. J Exp Med.

[41] Robinson DS, Larché M, Durham SR (2004). Tregs and allergic disease. J Clin Invest.

[42] Karagiannidis C, Akdis M, Holopainen P (2004). Glucocorticoids upregulate FOXP3 expression and regulatory T cells in asthma. J Allergy Clin Immunol.

